# Editorial: Noise-induced hearing loss: From basic to clinical research

**DOI:** 10.3389/fnint.2023.1172081

**Published:** 2023-03-17

**Authors:** Zhiwu Huang, Wei Qiu, Vicky Zhang, Hui Wang, Bin Ye, Qixuan Wang

**Affiliations:** ^1^Department of Otolaryngology Head and Neck Surgery, School of Medicine, Shanghai Ninth People's Hospital, Shanghai Jiao Tong University, Shanghai, China; ^2^College of Health Science and Technology, Shanghai Jiaotong University School of Medicine, Shanghai, China; ^3^State University of New York College at Plattsburgh, Plattsburgh, NY, United States; ^4^Department of Linguistics, Faculty of Human Sciences, Macquarie University, Sydney, NSW, Australia; ^5^National Acoustic Laboratories, Sydney, NSW, Australia; ^6^Department of Otolaryngology and Head and Neck Surgery, Shanghai Sixth People's Hospital, Shanghai Jiao Tong University, Shanghai, China; ^7^Department of Otolaryngology and Head and Neck Surgery, School of Medicine, Ruijin Hospital, Shanghai Jiao Tong University, Beijing, China

**Keywords:** noise exposure, hearing loss, tinnitus, cochlear synaptopathy, coding-in-noise deficits

Noise-induced hearing loss (NIHL) is one of the most common types of hearing loss among adults. The World Health Organization estimates that 10% of the world's population is exposed to sound levels that could potentially cause NIHL (Chadha et al., 2021). This Research Topic focused on NIHL was opened for submission from September 2021 to July 2022, with one opinion, four reviews, and eight original articles being included.

Exposure to industrial noise is one of the most common risks for NIHL. With the development of industrialization, non-Gaussian noise (also known as complex noise), which transients high-energy impulsive noise superimposed on the steady-state background noise, has been the primary noise type in the industry. Recent evidence showed that the temporal structure of complex noise could be expressed in the kurtosis metric (β), which is defined as the ratio of the fourth-order central moment to the squared second-order central moment of a distribution (Zhang et al., 2022a). Zhou et al. investigated the epidemiological characteristics of occupational NIHL among 1,050 manufacturing workers in China and found that kurtosis strengthens the association between noise exposure duration and noise intensity with high-frequency hearing loss. Shi et al. further validated the application of cumulative noise exposure (CNE) adjusted by kurtosis when evaluating occupational NIHL associated with non-Gaussian noise among 1,558 manufacturing workers from five industries in China. Their serial of studies demonstrated that the kurtosis-adjusted-CNE metric is more effective than CNE alone in assessing occupational NIHL among workers under non-Gaussian noise exposure. Recently, a draft guideline for measuring workplace noise exposure based on their work has been proposed in China (Zhang et al., 2022b).

NIHL is a complex condition with indiscernible mechanisms that result from exposure to loud sounds, and as research illustrates, is likely influenced by age, sex, genetics, underlying diseases, personal behaviors, and other physical and chemical hazards (Basner et al., 2014; Wang et al., 2021a). Chen et al. summarized primarily human studies as well as animal studies concerning the role of susceptible genes in NIHL, aims to provide insights into the further exploration of NIHL prevention and specific treatment. Meanwhile, Kurabi and team theorized several possible molecular pathways might be involved in NIHL (Kurabi et al., 2017). Zhao et al. focused on the adenylate-activated kinase (AMPK) pathway, and found that early AMPK activation may protect hearing by increasing ATP storage and reducing the release of large quantities of p-AMPK, which could help to inhibit synaptic damage.

Despite numerous investigations into NIHL, treatment options are still limited and preventive measures are not well implemented. NIHL can be avoided if appropriate preventive measures are adopted (The, 2019). Bramati et al. provided insights into the Dangerous Decibels^®^ program for the prevention of NIHL for noise-exposed workers. Their study showed greater effectiveness than the conventional educational-based intervention in a Brazilian population. In addition to occupational noise exposures, other noises may stem from everyday occurrences, and there are growing concerns about the increasing incidence of NIHL in children and adolescents who are potentially exposed to an array of loud sounds on a daily basis (Dillard et al., 2022). However, for non-occupational noise exposure, it is challenging to regulate as it would have to accommodate for the wide range of possible high-intensity sound sources, as there is high variability in activities that involve loud sounds for young people in their daily life. With the increasing application and contributions of neuroscience in recent NIHL studies, Pang and Gilliver proposed an opinion that neuroscience-informed approaches to reducing recreational NIHL for young people are required to meet the needs of the developing adolescent brain. Designing age-appropriate NIHL campaigns that consider these factors may increase the likelihood that interventions are efficacious and cost-effective.

Of late, several studies indicated that even moderate noise exposure could result in hearing difficulties in individuals with normal hearing thresholds, which has been referred to as “hidden hearing loss (HHL)” (Kohrman et al., 2020). Despite progress in pre-clinical models, evidence supporting the existence of HHL in humans remains inconclusive, and clinicians lack any non-invasive biomarkers that are sensitive to HHL (Bramhall et al., 2019; Wang et al., 2021b). Here, Valderrama et al. reviewed animal models of HHL as well as the ongoing research that aims to develop tools with which to diagnose and manage hearing difficulties associated with HHL. They discussed new research opportunities facilitated by recent methodological tools that may overcome a series of barriers that have hampered meaningful progress in diagnosing and treating of HHL.

Noise-induced synaptopathy (NIS) has been researched extensively as a potential cause of coding-in-noise deficits (CIND) and HHL. However, by using low-level, intermittent noise exposure mimicking the human experience in guinea pigs, Xia et al. found that degradations in signal processing were likely limited and not reflective of NIS and noise-induced HHL. Similarly, Pinsonnault-Skvarenina et al. also failed to find any significant association between noise exposure and auditory brainstem response outcomes, which might have detected cochlear synaptopathy in young factory workers with normal hearing. Ripley et al. further summarized the translational difficulties from animal data to human clinical, the technical challenges in quantifying NIS in humans, and the problems with the spontaneous rates theory on signal coding. The temporal fluctuation profile model was discussed as a potential alternative for signal coding at a high sound level against background noise, in association with the mechanisms of efferent control on the cochlea gain.

Cumulative damage from long-term noise exposure is also a major cause of age-related hearing loss, tinnitus, and even degraded learning and cognitive abilities (Manukyan, 2022). For noise-induced tinnitus, Hayes et al. developed the appetitive operant conditioning paradigm to assess acute and chronic sound-induced tinnitus in rats, which provides a platform for future investigations into the neural basis of tinnitus. For cognitive dysfunction related to noise exposure, Patel et al., exposed 6-month-old rats to an occupational-like noise and studied both hippocampal-dependent and striatal-dependent cognitive dysfunction. They highlighted that even mild noise exposure early in adulthood could have long-lasting implications for cognitive function later in life. Manohar et al. reviewed recent results that illustrate how NIHL deprives higher-order structures than the cochlea (such as the hippocampus) of the vital sensory information needed to carry out complex, higher-order functions.

We hope that this collection of articles on NIHL has provided readers with a comprehensive understanding of the current state of research in this area. Through the exploration of various influencing factors, mechanisms, prevention strategies, and non-auditory effects of NIHL, we have gained valuable insights into the complexities of this condition.

As we move forward, we encourage readers to use this information to guide their own research and clinical practices. Whether through the development of new prevention strategies or the advancement of early diagnosis and precise therapy, there is much work to be done in the NIHL area.

One key message that unites this entire collection is the importance of collaboration and interdisciplinary approaches to NIHL research. Only through the joint efforts of clinicians, scientists, engineers, and other stakeholders can we hope to make meaningful progress in our understanding and management of this population. We urge readers to join this effort and work toward a future where NIHL is a preventable and treatable condition.

## Author contributions

All authors except QW were guest editors of the Research Topic. QW was the research assistant and secretary on the Research Topic. All authors wrote the paper and approved the submitted version.
